# Association between liver fibrosis scores and short-term clinical outcomes in hospitalized chronic kidney disease patients: a prospective observational study

**DOI:** 10.3389/fmed.2024.1387472

**Published:** 2024-08-20

**Authors:** Dhriti Sundar Das, Anurag Anupam, Gautom Kumar Saharia

**Affiliations:** ^1^Department of General Medicine, All India Institute of Medical Sciences (AIIMS), Bhubaneswar, Odisha, India; ^2^Department of Biochemistry, AIIMS Bhubaneswar, Bhubaneswar, India

**Keywords:** chronic kidney disease, liver fibrosis, GPRI, clinical outcomes, observational study, association

## Abstract

**Introduction:**

In resource-constrained countries, inadequate access to healthcare and prognostic tools can be the Achilles’ heel in effectively managing chronic kidney disease (CKD). There is a significant similarity in the pathogenesis of CKD and liver fibrosis. The role of liver fibrosis (LF) scores in predicting short-term clinical outcomes in hospitalized patients with CKD is unknown. Our study aimed at calculating LF scores and studying the association of liver fibrosis with short-term mortality and morbidity in CKD patients.

**Methods:**

Patients aged above 15 years diagnosed with CKD as per the KDIGO criteria were enrolled. LF scores, namely, NFS, GPRI, and FIB-4 scores were calculated. Patients were followed up for a period of 28 days for good and poor composite outcomes, namely, the requirement of hemodialysis, non-invasive ventilation, prolonged hospital stay, and neurological and cardiovascular outcomes including death.

**Results:**

Among 163 patients, 70.5% were below 60 years of age, 82.2% were male and 35% were diabetic. At 28-day follow up, 52.1% had poor composite outcome. The AUROC for GPRI and FIB-4 in predicting poor outcomes was 0.783 (95% CI: 0.71–0.855) (*p* < 0.001) and 0.62 (95% CI: 0.534–0.706) (*p* = 0.008), respectively. The AUROC for GPRI and NFS in predicting all-cause mortality was 0.735 (95% CI: 0.627–0.843) (*p* = 0.001) and 0.876 (95% CI, 0.8–0.952) (*p* < 0.001), respectively.

**Conclusion:**

We found a positive association between LF scores and CKD outcomes in hospitalized patients. The LF scores significantly predicted poor outcomes in patients with CKD. Among the scores, GPRI was found to be a stronger predictor in predicting outcomes in both diabetic and non-diabetic patients with CKD. A high GPRI score was also associated with poor outcomes and increased mortality in both diabetics and non-diabetics.

## Highlights

- To the best of our knowledge, this is the only study to date that used liver fibrosis scores to study the short-term clinical outcome in CKD patients.
- In this study, multiple clinical outcomes were studied.
- Clinical outcomes were observed in both diabetics and non-diabetics and were evaluated separately.
- The study was conducted on a relatively small sample size of 163 patients.
- Although all liver disease patients were excluded from the study, underlying sub-clinical liver diseases cannot be ruled out, which may have affected the outcomes.

## Introduction

Chronic kidney disease (CKD) has become a prominent public health issue, with a significant impact on the healthcare economy (1). CKD entails structural damage to the kidneys and a gradual decrease in kidney function (a gradual fall in the rate of glomerular filtration rate), leading to kidney failure. Renal fibrosis is the end result of the continuing CKD phase, distinguished by the excessive buildup of extracellular matrix in the kidney tissues, leading to its shrinkage and a decline in organ functionality (2). CKD is linked to chronic inflammatory conditions and the initiation of pathways that promote fibrosis (2). The substantial impact of CKD arises not solely due to renal failure, which is connected to an increased likelihood of cardiovascular disease and a decrease in lifespan by 7–12 years (3). It is a complex ailment affecting multiple body systems, leading to increased morbidity and death rates, an elevated occurrence of cardiovascular incidents, and a weakened immune system, making individuals more susceptible to infections (4). This validates that CKD is linked to chronic inflammation and fibrosis in various parts of the body, including the liver (2). There is an unmet need to create cost-effective, dependable, consistent, and non-invasive scores for CKD outcomes.

It has been proven in various studies that liver fibrosis positively correlates with increased adverse events, morbidity, and mortality in patients suffering from diseases affecting various organ systems including but not limited to COPD, acute myocardial infarction, hemorrhagic transformation of ischemic stroke, hematoma volume, expansion of hematoma and mortality in hemorrhagic stroke, diabetes mellitus, and connective tissue disorders (5–9). This validates the theory that liver fibrosis is associated with chronic inflammation and fibrosis elsewhere in the body, i.e., the other organ systems, and hence carries a poorer prognosis in terms of all-cause morbidity and mortality as compared to a matched population without liver fibrosis.

However, cirrhosis of the liver also leads to renal dysfunction and a worse prognosis (10).

However, to this day, there has been no study that correlates LF scores with short-term clinical outcomes (in terms of morbidity and mortality) in hospitalized patients suffering from CKD. There is an unmet need to create cost-effective, dependable, consistent, and non-invasive scores for CKD outcomes. In resource-constrained healthcare settings, liver fibrosis (LF) scores present an apt alternative. Indeed, the use of a standardized system to evaluate the utility of biomarkers would facilitate their implementation in clinical practice. In this vein, the authors would like to report the use of GPRI, NFS, and FIB-4 scores as bedside predictor tools of all-cause morbidity and mortality in a heterogeneous population of hospitalized CKD patients for the first time. Therefore, this study was conducted to find the association between liver fibrosis as assessed by liver fibrosis scores and short-term clinical outcomes in CKD patients with the following objectives:

### Primary objectives

1. To estimate liver fibrosis (LF) scores (NFS, FIB-4, and GPRI) in hospitalized patients with chronic kidney disease.
2. To find out the association between LF scores and short-term clinical outcomes in these patients.

### Secondary objective

1. To find out the pattern of association between those with and without diabetes.

## Methods

Study design: A prospective observational study.
Setting: The study was conducted in the Department of General Medicine at the All India Institute of Medical Sciences, Bhubaneswar.
Study duration: February 2021 to September 2022.
Participants: Patients of age greater than or equal to 15 years admitted to the medicine inpatient ward with a presumptive diagnosis of CKD were screened and those fulfilling the selection criteria were included in the study.

### Definition of CKD

Chronic kidney disease is defined as “kidney damage or glomerular filtration rate (GFR) <60 mL/min/1.73 m^2^ for 3 months or more, irrespective of cause” (11).

### Stages of CKD

Stage 1: eGFR greater than 90 mL/min/1.73 m^2^ with kidney damage.
Stage 2: eGFR 60–89 mL/min/1.73 m^2^ with kidney damage.
Stage 3: eGFR 30–59 mL/min/1.73 m^2^ with kidney damage.
Stage 4: eGFR 15–29 mL/min/1.73 m^2^.
Stage 5: eGFR less than 15 mL/min/1.73 m^2^ or dialysis.

#### Inclusion criteria

Patients aged 15 years or older with a diagnosis of suspected or proven CKD as per the KDIGO guidelines willing to participate in the study and who did not meet any of the exclusion criteria.

#### Exclusion criteria

1. Patients diagnosed with chronic liver disease (known or clinically overt).
2. Patients with any malignancy.
3. Patients with hepatitis B or hepatitis C infection.
4. Patients with a history of liver transplant.
5. Pregnant patients.
6. Patients receiving drugs known to cause cirrhosis of liver.
7. Patients with co-existing infections likely to affect clinical outcomes.

### Sample size

Considering the incidence of liver fibrosis in CKD at 12% (12–14) and an expected response rate of 95% with 5% absolute precision and a 95% confidence interval, the sample size came out to be 171, which was rounded to 170 (15).

All patients admitted to AIIMS, Bhubaneswar, diagnosed to have CKD either by USG or as per KDIGO guidelines for diagnosis of CKD were screened for eligibility in this study. After obtaining proper consent, baseline laboratory investigations, i.e., complete blood count including an ESR, renal function test, and liver function test were performed. Chest X-ray, ultrasound of the abdomen, 12-lead ECG, 2D echocardiography, carotid Doppler, and non-contrast computed tomography of the brain were performed as and when required.

All liver function tests and CBC were performed, their height and weights were measured, their BMI was calculated, and their liver fibrosis scores, namely, NFS, GPRI, and FIB-4 scores were calculated by the formulae as mentioned below:

1. GPRI: It stands for GGT Platelet Ratio Index. GPRI = [(GGT/ULN OF GGT)]/[TPC] × 100.GPRI score ≥ 0.3 was considered significant (16).
2. FIB-4 Index = [Age x AST]/√[TPC per L × ALT]. FIB-4 score of greater than 1.45 [Ishak fibrosis stage 2–3] or more was considered significant (17).
3. NFS: It is a complex scoring system taking variables such as BMI, FBS, age, and albumin apart from liver enzymes. It is one of the most well-studied scoring systems.

A NAFLD fibrosis score of > −1.455 was considered significant for our study (18). Since short-term clinical outcomes were considered, patients were followed up for a period of 28 days either telephonically or via subsequent hospital visits or both. The outcomes were assessed by the treating clinician. The follow-up starts after the subjects were included in the study. The following clinical outcomes were taken into account.

### Outcome at follow-up

#### Good outcome

Good outcome poor composite outcome [maintenance hemodialysis (MHD), non-invasive ventilation (NIV), prolonged hospital stay, mechanical ventilation (MV), generalized tonic–clonic seizure (GTCS), stroke/myocardial infarction (MI), and death].

A good outcome was defined by a stable clinical course with no in-hospital deterioration as well as during follow-up. Poor composite outcomes, namely, MHD, NIV, prolonged hospital stay (more than 2 weeks), GTCS, stroke, and MI at follow-up were studied separately and as a composite entity.

### Data collection tools

All demographic profile, clinical history, signs and symptoms, laboratory investigation reports, treatment records, course in the hospital, and subsequent follow-up data up to 28 days were recorded in the predesigned case record form.

### Statistical analysis

The data were coded and recorded in the MS Excel spreadsheet program. SPSS v23 (IBM Corp.) was used for data analysis. Descriptive statistics were elaborated in the form of means/standard deviations and medians/inter-quartile range for continuous variables and frequencies and percentages for categorical variables. Group comparisons for continuously distributed data were made using an independent sample “*t*” test. If data were found to be non-normally distributed, appropriate non-parametric tests in the form of the Wilcoxon test were used. A chi-squared test was used for group comparisons of categorical data. In case the expected frequency in the contingency tables was found to be <5 for >25% of the cells, the Fisher’s exact test was used instead. Statistical significance was kept at *p* < 0.05.

Receiver operator curve analysis along with sensitivity and specificity was performed to predict an optimal cut-off for a continuous predictor predicting a binary outcome. Logistic regression analysis was performed for multivariable adjustment to fully characterize the association between the baseline variables and the outcome. The variables for the model were selected on the basis of significant and most useful variables in the univariable model.

### Ethical consideration

A voluntary written informed consent was obtained from each patient and their attendant. The benefit and harm of joining the study and the freedom of withdrawing from the study at any moment were explained to them. The study was conducted following ICMR’s ethical guidelines for biomedical research on human subjects (2006) after getting written permission from the institutional ethics committee.

## Results

**Figure 1:**
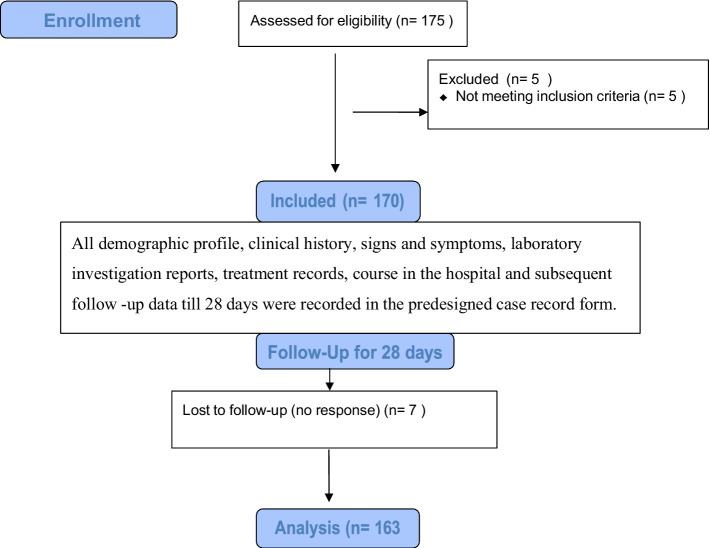
Flow diagram.

A total of 175 CKD patients were initially enrolled in the study; of these, five were excluded from the study as they were found to have chronic hepatitis B on medical record review, meeting the exclusion criteria for this study. Seven patients were lost to follow-up. Finally, 163 participants with a diagnosis of CKD according to the KDIGO criteria were admitted to AIIMS, Bhubaneswar, and followed up for 28 days to study the relationship between liver fibrosis scores, namely, GPRI, FIB-4, and NFS and their short-term composite outcomes. Overall, 22.7% of the participants were end-stage renal disease (ESRD) (eGFR<15 mL/min) patients on MHD (Figure 1).

**Figure 2:**
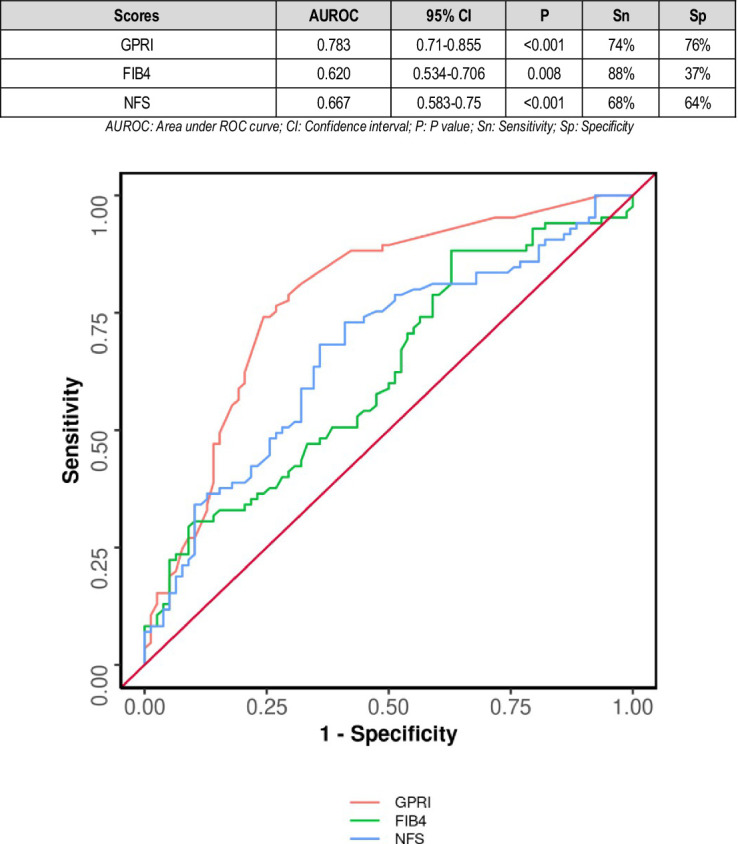
Comparison of the performance of various scores in predicting poor composite outcome.

The area under the ROC curve (AUROC) for GPRI in predicting poor outcomes at follow-up was 0.783 (95% CI: 0.71–0.855), thus demonstrating fair diagnostic performance. It was statistically significant (*p* = 0.011). A cutoff of GPRI ≥0.4 predicted poor outcomes with a sensitivity of 80% and a specificity of 61%. The AUROC for FIB-4 in predicting poor outcomes was 0.62 (95% CI: 0.534–0.706) with satisfactory diagnostic performance (*p* = 0.008). A cutoff of FIB-4 ≥ 0.5 predicted poor outcome with a sensitivity of 88% and a specificity of 37% (Figure 2).

**Figure 3:**
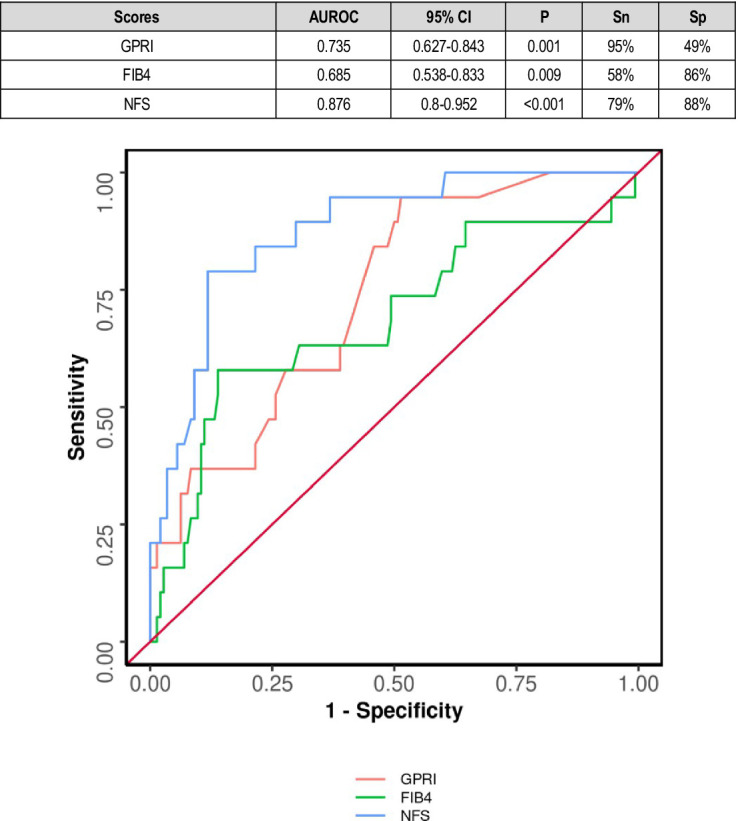
Comparison of the performance of various scores in predicting death.

The area under the ROC curve (AUROC) for GPRI in predicting mortality was 0.735 (95% CI: 0.627–0.843) (*p* = 0.001). A cutoff of GPRI ≥0.32 predicted death with a sensitivity of 95% and a specificity of 49%. The area under the ROC curve (AUROC) for FIB-4 in predicting mortality was 0.685 (95% CI: 0.538–0.833), thus demonstrating poor diagnostic performance. It was statistically significant (*p* = 0.009). A cutoff of FIB-4 ≥ 1.86 predicted death with a sensitivity of 58% and a specificity of 86% (Figure 3).

**Figure 4:**
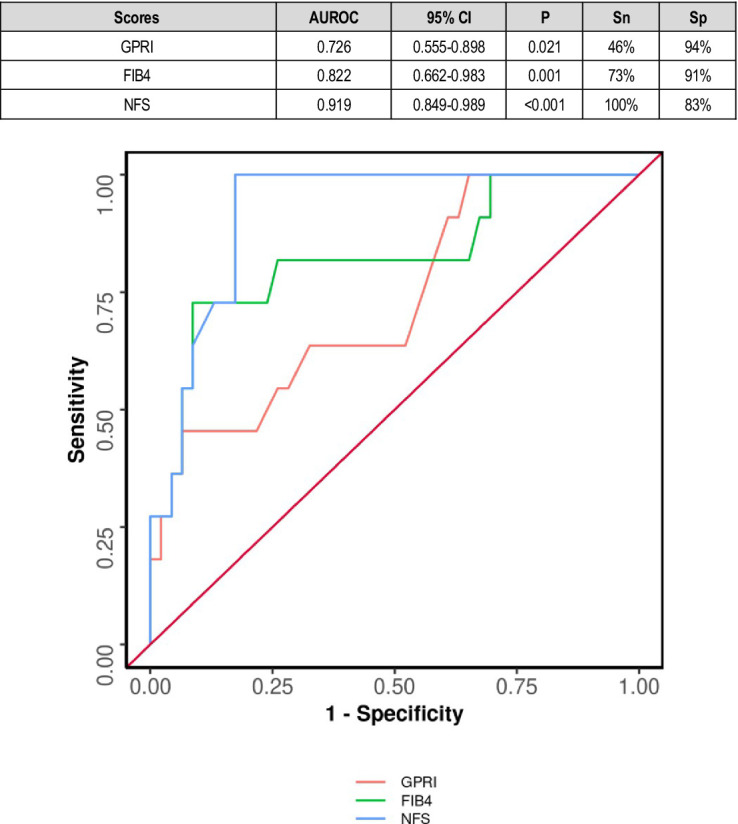
Comparison of the performance of various scores in predicting death in diabetes subjects.

In diabetics, the area under the ROC curve (AUROC) for GPRI in predicting death was 0.726 (95% CI: 0.555–0.898) (*p* = 0.021). A cutoff of GPRI ≥1.4 predicted death with a sensitivity of 46% and a specificity of 94% (Figure 4).

**Figure 5:**
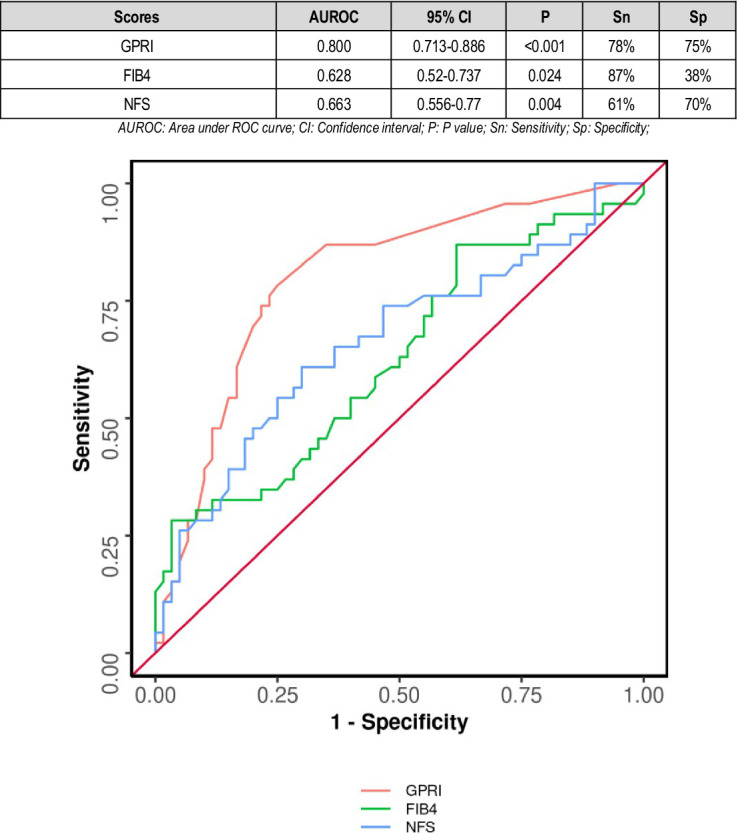
Comparison of the performance of various scores in predicting poor composite outcome in non-diabetics.

The diagnostic performance of GPRI (AUC = 0.800) was significantly better than that of NFS (AUC = 0.663) (DeLong's test *p* = 0.020). The diagnostic performance of GPRI (AUC = 0.800) was significantly better than that of FIB-4 (AUC = 0.628) (DeLong's test *p* = 0.001). There was no significant difference in the diagnostic performance of NFS and FIB-4 (DeLong's test *p* = 0.570) (Figure 5).

**Figure 6:**
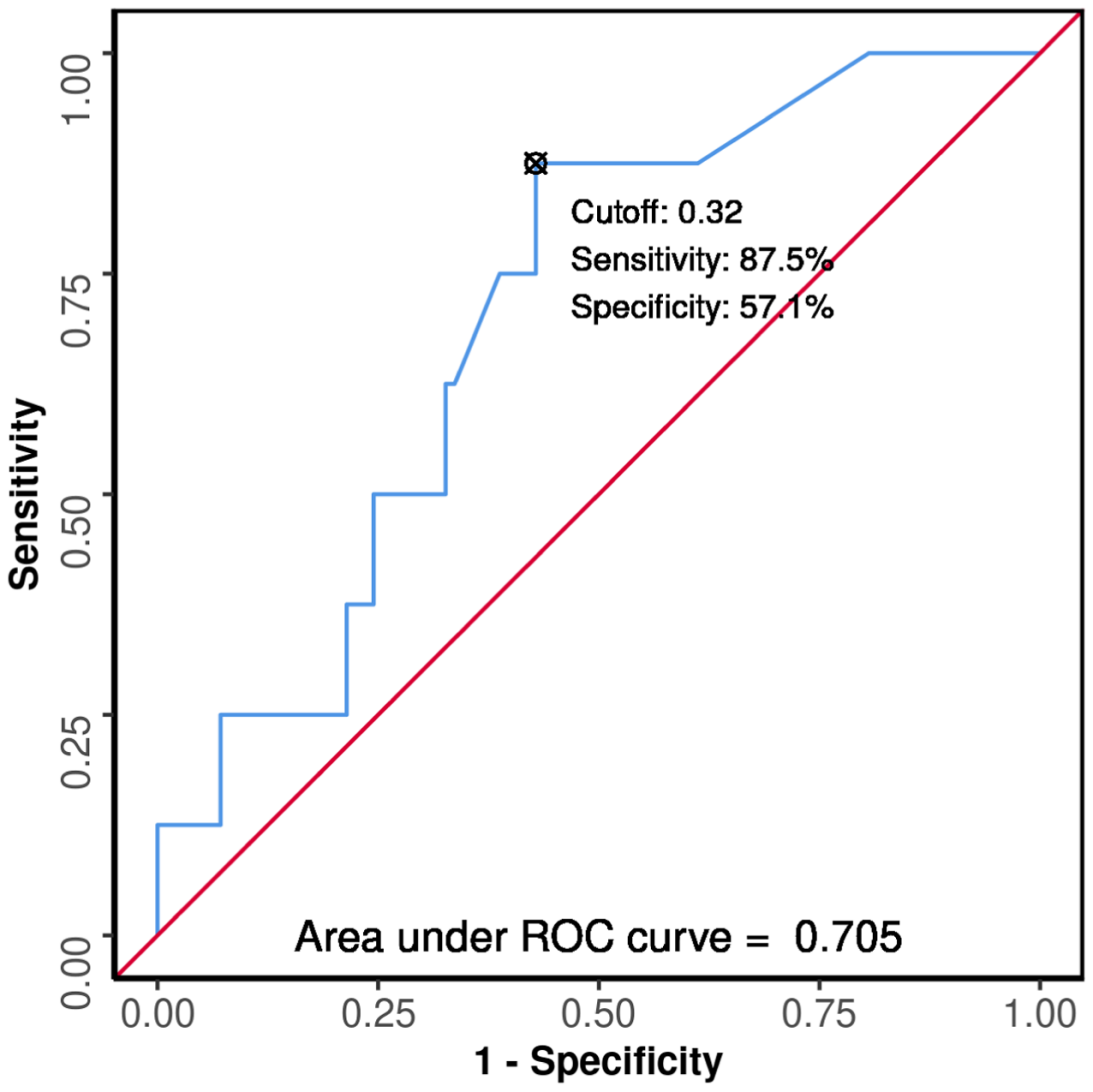
ROC curve analysis showing performance of GPRI in predicting mortality in non-diabetics.

In non-diabetics, the area under the ROC curve (AUROC) for GPRI predicting death was 0.705 (95% CI: 0.541–0.87), thus demonstrating fair diagnostic performance (*p* = 0.054). A cutoff of GPRI ≥0.32 predicted death with a sensitivity of 88% and a specificity of 57% (Figure 6).

### Regression with selected variables in model

As shown in the Table 1, “Bidirectional Stepwise Selection” is used to select only the most useful variables to include in the final multivariable predictive model for the dependent variable.

**Table 1:** Regression with selected variables in model.

**Dependent: Outcome**		**Good**	**Poor**	**OR (univariable)**	**OR (multivariable)**
Age (Years)	Mean (SD)	51.2 (14.8)	50.8 (16.9)	1.00 (0.98–1.02, *p* = 0.886)	0.97 (0.95–1.01, *p* = 0.108)
Gender	Men	67 (50.0)	67 (50.0)	-	-
	Women	11 (37.9)	18 (62.1)	1.64 (0.72–3.73, *p* = 0.241)	-
Diabetic Status	Diabetic	18 (31.6)	39 (68.4)	-	-
	Non-diabetic	60 (56.6)	46 (43.4)	0.35 (0.18–0.70, *p* = 0.003)	0.40 (0.15–1.08, *p* = 0.071)
HTN	No	37 (82.2)	8 (17.8)	-	-
	Yes	41 (34.7)	77 (65.3)	8.69 (3.70–20.38, *p* < 0.001)	12.65 (3.82–41.90, *p* < 0.001)
Dyslipidemia	No	60 (55.6)	48 (44.4)	-	-
	Yes	18 (32.7)	37 (67.3)	2.57 (1.30–5.07, *p* = 0.006)	-
GPRI	Mean (SD)	0.4 (0.5)	1.0 (1.4)	4.55 (2.01–10.32, *p* < 0.001)	4.75 (2.00–11.32, *p* < 0.001)
FIB-4	Mean (SD)	1.0 (0.7)	1.3 (1.0)	1.66 (1.13–2.44, *p* = 0.010)	-
NFS	Mean (SD)	−1.1 (1.4)	−0.2 (1.6)	1.58 (1.22–2.04, p < 0.001)	1.74 (1.21–2.49, p = 0.003)
CKD stage	I	11 (44.0)	14 (56.0)	-	-
	II	31 (60.8)	20 (39.2)	0.51 (0.19–1.34, *p* = 0.170)	0.30 (0.08–1.14, *p* = 0.076)
	III	25 (65.8)	13 (34.2)	0.41 (0.15–1.15, *p* = 0.090)	0.33 (0.08–1.40, *p* = 0.132)
	IV	7 (58.3)	5 (41.7)	0.56 (0.14–2.26, *p* = 0.416)	0.86 (0.13–5.87, *p* = 0.881)
	V	4 (10.8)	33 (89.2)	6.48 (1.76–23.88, *p* = 0.005)	2.89 (0.59–14.14, *p* = 0.190)

Model fit: χ^2^(9) = 89.36, *p* ≤ 0.001 Pseudo-*R*^2^ = 0.4.

Number in dataframe = 163, Number in model = 163, Missing = 0 AIC = 156.3, *C*-statistic = 0.889, H&L = Chi-sq (8) 4.52 (*p* = 0.807).

## Discussion

Chronic kidney disease is not merely the progressive loss of renal function and accumulation of toxic nitrogenous wastes in the body; it is a multisystem disease with widespread deleterious effects on all organ systems and metabolism. It has a devastating impact in terms of mortality and morbidity in patients, more so in low- and middle-income countries. Furthermore, the burden of malnutrition and poor sanitary conditions predisposing to infections and limited access to dialysis and other supportive treatments complicate the management of kidney health.

There is a lack of prognostic markers in CKD beyond conventional renal function tests and the estimated glomerular filtration rate, which predict outcomes in chronic kidney disease patients. Non-invasive liver fibrosis scores rely on various parameters such as liver transaminases, platelet count, BMI, and glycemic status, are a fairly good surrogate of liver fibrosis, and can substitute FibroScan in resource-poor settings. Not only have they been successfully used to diagnose liver fibrosis and poor outcomes in diseases such as hepatitis B, hepatitis C, and NAFLD but also in unrelated systemic diseases.

Multiple studies in the recent past have studied the non-invasive liver fibrosis scores as outcome/prognostic markers in various unrelated systemic diseases such as stroke, COPD, and sepsis. In this study, 47.9% of patients had good outcomes, while 52.1% of patients had poor outcomes at 28-day follow-up (Table 2).

**Table 2:** Clinical characteristics of the participants.

Variables	All study participants (*n* = 163)	Good outcome (*n* = 78)	Poor composite outcome (*n* = 85)	*p*-value
**Age (Years)**	51.01 ± 15.87	51.19 ± 14.84	50.84 ± 16.86	0.886^1^
**Gender**				0.238^3^
Men	134 (82.2%)	67 (85.9%)	67 (78.8%)	
Women	29 (17.8%)	11 (14.1%)	18 (21.2%)	
**Hypertension** ^***^	118 (72.4%)	41 (52.6%)	77 (90.6%)	<0.001^3^
**Thyroid status** ^***^				0.005^2^
Euthyroid	130 (79.8%)	69 (88.5%)	61 (71.8%)	
Hypothyroid	32 (19.6%)	8 (10.3%)	24 (28.2%)	
Hyperthyroid	1 (0.6%)	1 (1.3%)	0 (0.0%)	
**Dyslipidemia** ^***^	55 (33.7%)	18 (23.1%)	37 (43.5%)	0.006^3^
**BMI (Kg/m** ^2^ **)**	22.28 ± 3.26	22.02 ± 2.98	22.52 ± 3.50	0.614^4^
**Diabetic status** ^***^				0.002^3^
Diabetic	57 (35%)	18 (23.1%)	39 (45.9%)	
Non-diabetic	106 (65%)	60 (76.9%)	46 (54.1%)	
**CKD stage** ^***^				<0.001^3^
I	25 (15.3%)	11 (14.1%)	14 (16.5%)	
II	51 (31.3%)	31 (39.7%)	20 (23.5%)	
III	38 (23.3%)	25 (32.1%)	13 (15.3%)	
IV	12 (7.4%)	7 (9.0%)	5 (5.9%)	
V	37 (22.7%)	4 (5.1%)	33 (38.8%)	
**GPRI** ^***^	0.68 ± 1.11	0.38 ± 0.47	0.98 ± 1.42	<0.001^4^
**GPRI category** ^***^				<0.001^3^
≤0.3	69 (42.3%)	53 (67.9%)	16 (18.8%)	
>0.3	94 (57.7%)	25 (32.1%)	69 (81.2%)	
**FIB-4** ^***^	1.146 ± 0.89	0.95 ± 0.66	1.32 ± 1.04	0.008^4^
**FIB-4 category**				0.094^3^
≤1.45	113 (69.3%)	59 (75.6%)	54 (63.5%)	
>1.45	50 (30.7%)	19 (24.4%)	31 (36.5%)	
**NFS** ^***^	−0.59 ± 1.56	−1.07 ± 1.41	−0.16 ± 1.58	<0.001^4^
**NFS category**				0.104^3^
≤ − 1.455	35 (21.5%)	21 (26.9%)	14 (16.5%)	
> − 1.455	128 (78.5%)	57 (73.1%)	71 (83.5%)	

^***^Significant at *p* < 0.05, 1: *t*-test, 2: Fisher’s exact test, 3: Chi-squared test, 4: Wilcoxon–Mann–Whitney U-test.

According to a study by Xianghua Zeng and others in 2015, higher GPRI scores were associated with poorer outcomes in hepatitis B patients (19). In our study, the area under the ROC curve (AUROC) for GPRI in predicting poor outcomes at follow-up was 0.783 (95% CI: 0.71–0.855), thus demonstrating fair diagnostic performance. It was statistically significant (*p* = 0.011) (Figure 2).

High GPRI was strongly associated with poor outcomes in both diabetics and non-diabetics. High GPRI was also associated with increased mortality in both diabetics and non-diabetics. GPRI was significant in predicting poor outcomes, prolonged hospitalization, and death. There was no significant difference in its performance among diabetic and non-diabetic groups.

Fibrosis-4 scores were used for predicting outcomes in the same group of patients. In a study by Yuan et al. (5), the FIB-4 score was associated with hemorrhagic transformation in ischemic stroke. In another study by Zhu et al. (9), the FIB-4 score had a direct correlation with poor outcomes in sepsis. In a study by Liu et al. (20), the FIB-4 score had a 1.57–1.92-fold increased risk of primary end points in terms of cardiovascular outcome in patients treated with PCI, *p* value less than 0.001. In our study, the odds ratio of having poor outcomes in those with a FIB-4 score > 1.45 was 1.3 (0.95–1.72), which was consistent with the results of the aforesaid study (Table 1).

As seen from the results, FIB-4 had poor performance in predicting outcomes in diabetics. This may be attributed to additional factors involved in diabetics such as glycemic control and other complications of diabetes. Overall, high FIB-4 scores were associated with increased mortality, as evident in other studies in both diabetics and non-diabetics, which was consistent with the findings of previous studies.

In total, 55% of patients with significant fibrosis as per the NFS score had poor outcomes as compared to 40% of those without significant fibrosis. However, the difference was not statistically significant (Table 2). In a study by Chen et al. (8), LF scores including NFS were associated with increased mortality in CAD patients.

The area under the ROC curve (AUROC) for NFS in predicting death was 0.876 (95% CI: 0.8–0.952), thus demonstrating good diagnostic performance. It was statistically significant (*p* ≤ 0.001). A cutoff of NFS ≥0.58 predicted mortality with a sensitivity of 79% and a specificity of 88% (Figure 3).

NAFLD fibrosis score was a relatively poor predictor of outcome in diabetics. This might be explained by other factors in diabetes including immuno-suppression and micro- and macro-vascular complications other than nephropathy contributing to outcomes. However, it was an excellent predictor of mortality in diabetic patients, which was consistent with other studies (Figure 4).

**Figure 7:**
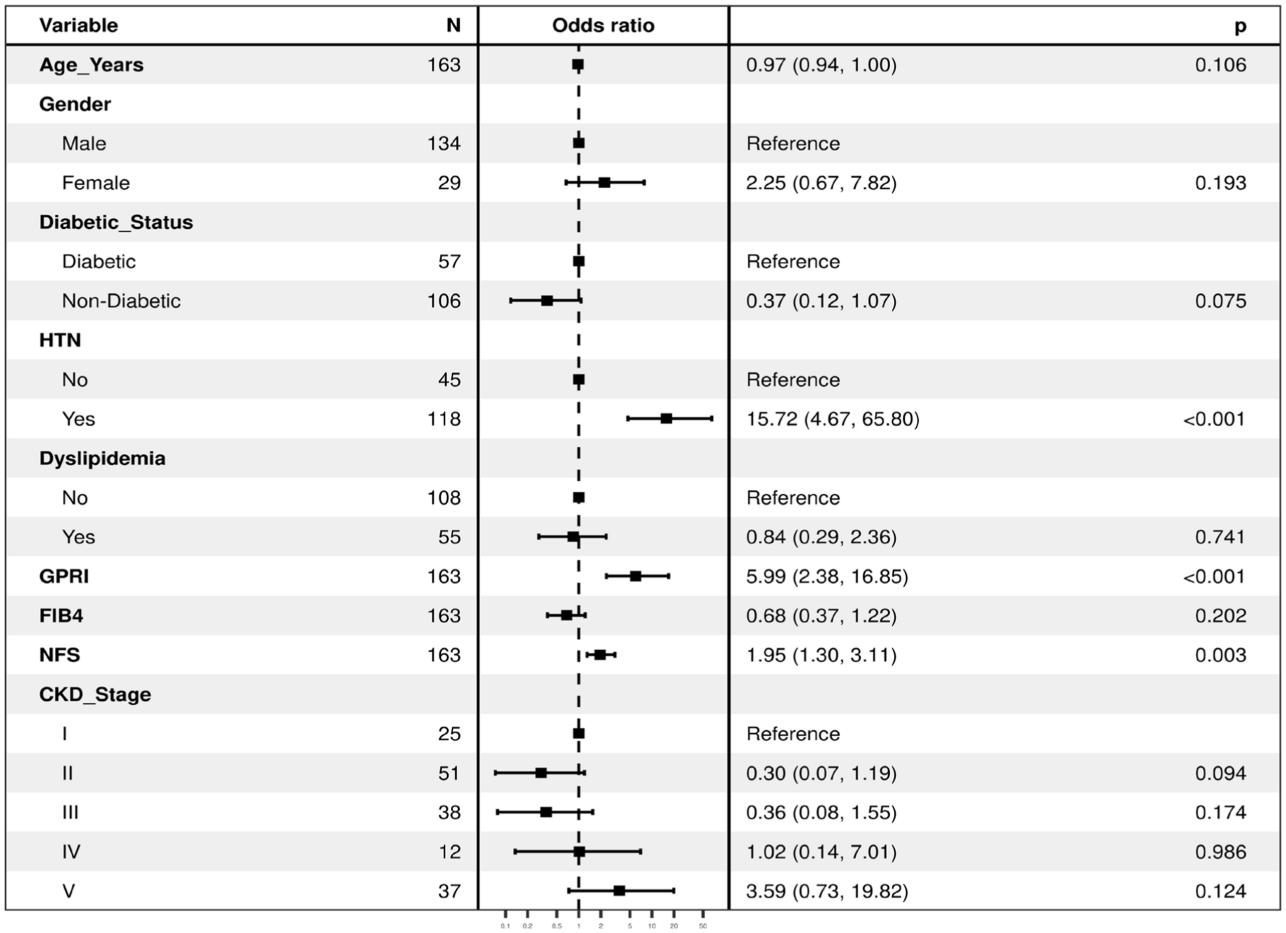
The regression analysis for the dependent variable (outcome) using all the predictor variables together in one go (with all the variables in the regression model). It lists the odds ratios for each of the variables with respect to the outcome when they are entered in the model together as predictors of the dependent variable (thus controlling for each other). The first category in each of the categorical variables is the reference category, against which the odds ratios of the rest of the variables are calculated. Model Fit: χ²(12) = 92.8, *p* ≤ 0.001 Pseudo-*R*² = 0.41. Number in dataframe = 163, Number in model = 163, AIC = 158.9, C-statistic = 0.894, H&L = Chi-sq (8) 5.19 (*p* = 0.737).

Overall, all liver fibrosis scores were associated with adverse outcomes in both diabetics and non-diabetics. GPRI was the best in terms of predicting poor outcomes in diabetics and non-diabetics, whereas NFS was the best at predicting mortality in diabetic patients even after adjustment of the baseline factors in the regression analysis (Table 1; Figure 7).

Liver fibrosis scores initially crafted for predicting fibrosis in NAFLD and viral hepatitis have recently come into the limelight as a prognostic tool after various studies in non-hepatological diseases. Studies have proved that they predicted poor clinical outcomes in these patients independent of any hepatic involvement. Although renal function tests and other methods of estimation of glomerular filtration rate reflect the functional status of the kidney and outcomes stemming from renal compromise, they do not necessarily always translate into poorer clinical outcomes. Liver fibrosis scores can be a novel strategy and an inexpensive and readily available prognostication tool at the bedside for clinicians. Diabetes and dyslipidemia were quite common in the study population, and therefore the associated underlying metabolic dysfunction-associated fatty liver disease (MAFLD) may highlight the prognostic importance of blood biomarkers studied. MAFLD has been increasingly recognized as a systemic disease with increased cardiovascular events and cancers (10). This study however excluded patients with known chronic liver disease or clinically overt liver disease. However, underlying sub-clinical liver disease still remains a concern and may be a potential limitation of the study.

Possible mechanisms for the association of liver fibrosis biomarkers with chronic kidney disease may be explained in the following ways: First, sub-clinical liver fibrosis in this cohort of patient, which included diabetes and dyslipidemia may account for the observed association. Second, potential explanation for the association of liver biomarkers predicting adverse outcomes of non-hepatic diseases may be due to extra hepatic fibrosis as CKD is linked with inflammation and fibrosis in other organs including the liver, impacting the biomarkers estimation (2). Third, oxidative stress remains a crucial common pathophysiological entity among liver disease and chronic kidney disease (21). Previous studies have shown that oxygen radicals and other prooxidant factors may play a decisive role in the complications of chronic kidney injury (21). Finally, circulating platelet-derived growth factor (PDGF-D) may account for its significant role in the fibrogenesis of the liver and kidneys (22). In addition, leptin, an adipokine, is also involved in the profibrotic process of both the liver and the kidneys (23).

## Strengths and limitations of the study

To the best of our knowledge, this is the only study to date that used liver fibrosis scores to study the outcome in CKD patients. In this study, multiple clinical outcomes were studied, and both diabetics and non-diabetics were evaluated separately.

The study was conducted on a relatively small sample size of 163 patients. Although all known or clinically overt liver disease patients were excluded from the study, underlying sub-clinical liver diseases including fatty liver disease cannot be ruled out, which may have affected the outcomes. Furthermore, other non-invasive methods of liver fibrosis such as baseline FibroScan were not available in the cohort for ruling out significant liver disease. Moreover, prolonged hospital stay may not have the same level of clinical severity as the other outcomes mentioned. It is more reflective of the healthcare system’s burden and the impact on the patient’s quality of life during the hospitalization period.

Overall, all liver fibrosis scores were associated with adverse outcomes in both diabetic and non-diabetic CKD patients. GPRI was the best in terms of predicting outcome in both diabetic and non-diabetic CKD patients, whereas NFS was the best at predicting mortality in diabetic CKD patients. However, it is to be stated that these scores would need validation in other cohorts for this clinical setting before it is used in clinical practice.

## Conclusion

Liver fibrosis calculated with non-invasive LF scores was associated with higher short-term mortality and morbidity among CKD patients, whereas there was no significant difference in the outcomes seen among diabetics and non-diabetics. Liver fibrosis scores are a reliable predictor of outcome in CKD patients, can be used as a prognostication tool for CKD patients, and urge further research for risk stratification in such cohort of individuals.

## Data availability statement

The raw data supporting the conclusions of this article will be made available by the authors, without undue reservation.

## Ethics statement

The studies involving humans were approved by Institution Ethics Committee, AIIMS Bhubaneswar. The studies were conducted in accordance with the local legislation and institutional requirements. The participants provided their written informed consent to participate in this study.

## Author contributions

DD: Conceptualization, Data curation, Formal analysis, Investigation, Methodology, Project administration, Resources, Software, Supervision, Validation, Visualization, Writing – original draft, Writing – review & editing. AA: Data curation, Formal analysis, Validation, Writing – original draft, Writing – review & editing. GS: Investigation, Writing – review & editing.

## Glossary

AAR: Aspartate-to-alanine aminotransferase ratio
APRI: AST Platelet Ratio Index
API: Age platelet index
AST: Aspartate transaminase
ALT: Alanine transaminase
AMI: Acute myocardial infarction
AUC: Area under curve
AUROC: Area under ROC
BMI: Body mass index
CAD: Coronary artery disease
COPD: Chronic obstructive pulmonary disease
CKD: Chronic kidney disease
ECM: Extracellular matrix
ESR: Erythrocyte sedimentation rate
FIB-4: Fibrosis-4 Score
Fibro-Q: Fibro-quotient
GI: Gastrointestinal
GPRI: GGT Platelet Ratio Index
GGT: Gamma-glutamyl transferase
GTCS: Generalized tonic–clonic seizures
HD: Hemodialysis
ICH: Intra-cerebral hemorrhage
ICU: Intensive care unit
ICMR: Indian Council of Medical Research
KDIGO: Kidney disease improving global outcomes
LF SCORE: Liver fibrosis score
LSM: Liver stiffness measure
MHD: Maintenance hemodialysis
MV: Mechanical ventilation
NFS: NAFLD fibrosis score
NIV: Non-invasive ventilation
NAFLD: Non-alcoholic fatty liver disease
ROC: Receiver operator curve
TGF: Transforming growth factor
TPC: Total platelet count
